# *Aspergillus niger* PA2 Tyrosinase Covalently Immobilized on a Novel Eco-Friendly Bio-Composite of Chitosan-Gelatin and Its Evaluation for L-DOPA Production

**DOI:** 10.3389/fmicb.2016.02088

**Published:** 2016-12-22

**Authors:** Pragati Agarwal, Swati Dubey, Mukta Singh, Rajesh P. Singh

**Affiliations:** Department of Biotechnology, Indian Institute of Technology RoorkeeRoorkee, India

**Keywords:** Immobilization, tyrosinase, chitosan, gelatin, L-DOPA

## Abstract

Tyrosinase (EC 1.14.18.1) a copper-containing monooxygenase, isolated from a fungal isolate *Aspergillus niger* PA2 was subjected for immobilization onto a composite consisting of chitosan and gelatin biopolymers. The homogeneity of the chitosan-gelatin biocomposite film was characterized by X-ray diffraction analyses. To evaluate immobilization efficiency, chitosan-gelatin-Tyr bio-composite films were analyzed by field emission scanning electron microscopy, atomic force microscopy and UV-spectroscopy. The rough morphology of the film led to a high loading of enzyme and it could retain its bioactivity for a longer period. The enzyme adsorbed onto the film exhibited 72% of its activity after 10 days and exhibited good repeatability for up to nine times, after intermittent storage. Moreover, the immobilized enzyme exhibited broader pH and temperature profile as compared to free counterpart. Immobilized enzyme was further evaluated for the synthesis of L-DOPA (2,4-dihydroxy phenylalanine) which is a precursor of dopamine and a potent drug for the treatment of Parkinson's disease and for myocardium neurogenic injury.

## Introduction

Enzymes have enormous potential to be used as catalysts in a wide range of biotechnological and industrial processes. They offer a distinct advantage due to their specificity, high catalytic efficiency and biodegradability (Bullock, 1995; Munjal and Sawhney, 2002). A common drawback of enzymatic procedures is the consumption of expensive enzyme molecules during the process (Fiorentino et al., 2010). Thus, the industrial exploitation of enzymes is limited due to their non-reusability and instability (Wang et al., 2009). Enzyme immobilization has been found to be a promising way to overcome these limitations and to enhance the catalytic performance of enzymes (Ensuncho et al., 2005; Arica and Bayramoglu, 2006; Bayramoglu et al., 2012). Immobilization of enzyme has several advantages, such as improved stability by protecting the protein from deactivation, high reproducibility, improved thermal and storage stability, significant reduction in the production cost, easy recovery of the enzyme and continuous and repeated use (Cirpan et al., 2003; Yildiz et al., 2013).

Polymer blending is one of the most productive approaches for providing novel and desirable polymeric materials to immobilize enzymes (Tembe et al., 2006). The composite thus prepared, combine the physicochemical attributes of both components and overcome their shortcomings (Gupte and D'Souza, 1999; Jha and D'Souza, 2005). Also this simply-prepared protein–polysaccharide bio-composite film facilitates the enzymes to retain their activity (Forzani et al., 1999, 2000). Biocompatible polymers have their unique advantages for enzyme immobilization, as they aid in retaining the activity of enzyme well by providing desirable micro-environment (Li et al., 2006). Chitosan (2-acetamido-2-deoxy-d-glucose) is an N-deacetylated derivative with chemically modified acetyl group of chitin. Chitosan is widely considered to be a biocompatible, nontoxic and chemically distinctive polysaccharide which occurs as a natural biopolymer in the exoskeleton of crustaceans and in fungal cell wall (Liu et al., 2005). Due to its unique chemical properties, such as excellent membrane-forming ability, high permeability toward water, good adhesion, biocompatibility, non-toxicity, biodegradability, high mechanical strength and susceptibility to chemical modifications due to the presence of reactive amino and hydroxyl functional groups (Cruz et al., 2000), chitosan serves as a matrix for the assembly of biomolecules including enzymes, DNA, and antibodies (Ye et al., 2005; Tan et al., 2009; Xu et al., 2010). Gelatin is also a naturally occurring biopolymer having good adhesion qualities and non-toxicity. It is commonly used as a gelling agent in food, such as marsh mallows, desserts, ice-creams and pharmaceuticals. Also it is commonly used as a biological substrate to culture adherent cells. The composite film consisting of chitosan and gelatin mixed and gelled together, can overcome the brittleness of pure chitosan matrix and its shrinkage. Another advantage of this blend is that both the polymers are biocompatible, transparent, safe, abundant and inexpensive, thereby providing substantial microenvironment for enzyme to be immobilized.

Tyrosinase is a copper containing multifunctional metalloenzyme that catalyzes the hydroxylation of monophenols to *o*-diphenols (monophenolase or cresolase activity) and the oxidation of the latter to *o*-quinones (diphenolase or catecholase activity), using oxygen (Labus et al., 2011). The two activities can be used as the basis for widespread biotechnological and industrial applications, such as biosensors to determine the phenolic content in waste water (Atlow et al., 1984), for bioremediation of phenolic effluents and for biotransformation of L-tyrosine to L-DOPA, the preferred drug for Parkinson's disease.

Previously chitosan-gelatin composite formation has been studied by Chen et al. (2003), Basavaraju et al. (2006), and Mi (2005) and its application for food products had been explored by Arvanitoyannis et al. (1998) and Lo'pez-Caballero et al. (2005), but this bio-composite had never been investigated to be used as a matrix for tyrosinase immobilization. However, several workers have endeavored to immobilize tyrosinase onto alginate, polyacrylamide and gelatin gels (Munjal and Sawhney, 2002), ZnO nanoparticles (Li et al., 2006), cellulose support (Labus et al., 2011) and polystyrene microplate (Saini et al., 2014). Moreover, this is the first report of immobilization of tyrosinase from *A. niger* on any support matrix.

**Figure 1:**
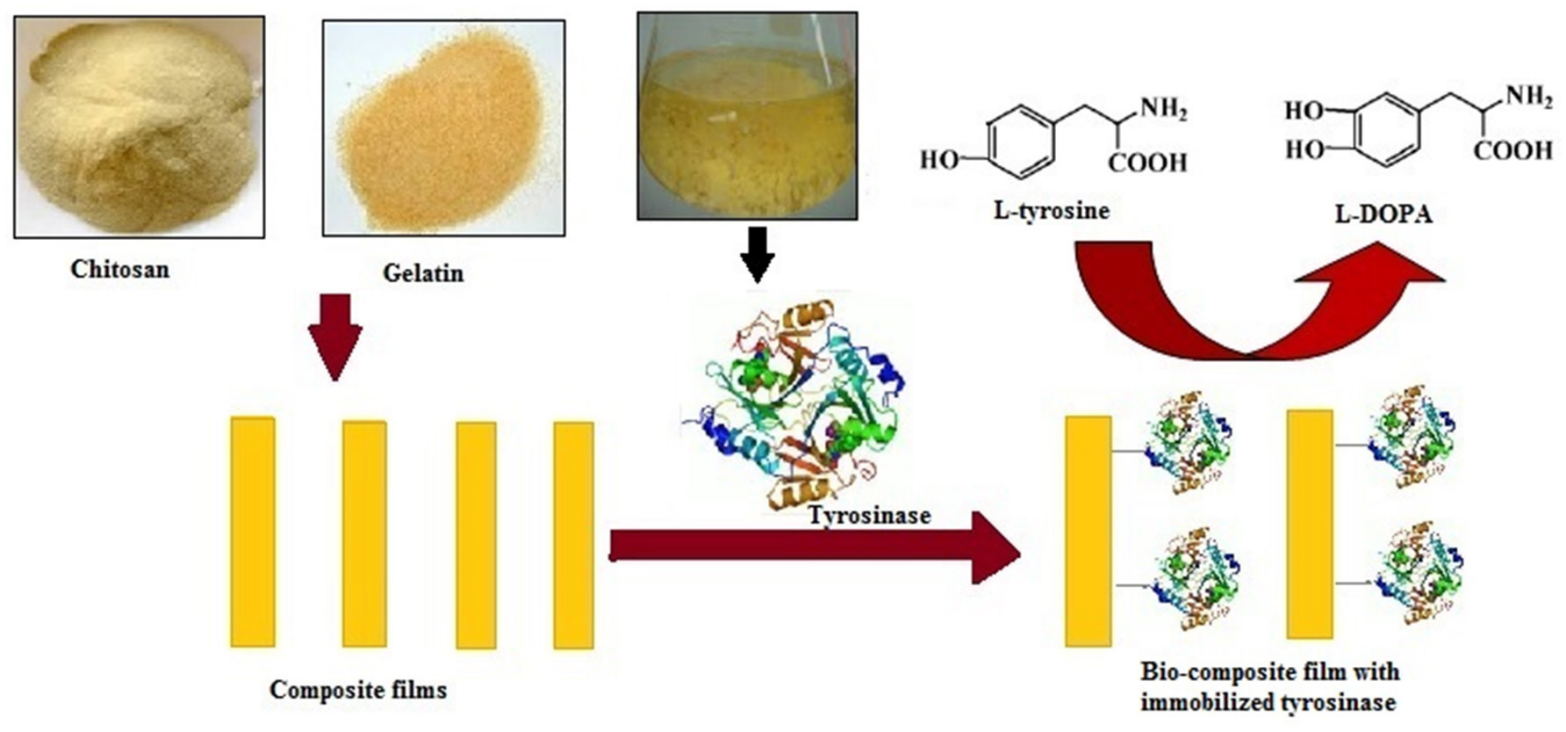
**Schematic representation of immobilization of *Aspergillus niger* PA2 tyrosinase onto chitosan-gelatin composite film and L-DOPA production**.

In the present study, tyrosinase from *A. niger* PA2 had been immobilized onto a bio-composite film consisting of chitosan and gelatin polymers (Figure 1). The prepared films were thoroughly characterized using XRD, FESEM, AFM, and UV-spectroscopy and the efficiency of immobilized tyrosinase was evaluated in terms of stability, reusability and for the production of L-DOPA.

## Materials and methods

### Materials

Chitosan and gelatin were procured from Sigma–Aldrich (India). All other chemicals bought were of analytical grade. Solutions and reagents were prepared using Milli-Q water.

### Enzyme preparation

For the production of tyrosinase, *A. niger* PA2 isolated from waste water effluent (Agarwal et al., 2016) was grown in Vogel's medium (Vogel, 1956) at 30°C (150 rpm) for 5–6 days. The supernatant from the culture was filtered using cheese cloth and the filtrate thus obtained was centrifuged at 6000 rpm for 15 min and the supernatant was used as the crude preparation. The crude preparation was further subjected to ammonium sulfate fractionation (25–75%). Briefly, for each saturation ammonium sulfate was added to the above preparation and the mixture was stirred for 1 h at 4°C and then centrifuged (10,000 rpm, 45 min). Supernatant obtained was further added with ammonium sulfate so as to achieve further saturation. The precipitate obtained was collected and redissolved in 20 ml of fresh extraction buffer (50 mM phosphate buffer, pH 6.5) and dialyzed against the same buffer for 10 h at 4°C. The concentrated enzyme thus obtained was stored at 4°C (Munjal and Sawhney, 2002; Saini et al., 2014).

### Composite film preparation

Acidic solutions of chitosan (2%, 2 ml) and gelatin (2%, 2 ml) were prepared separately by dissolving in acetic acid (1%, v/v) and then were mixed together in 1:1 proportion. This mixture was stirred for 4 h at room temperature and the homogeneous blend was then spread uniformly on a glass plate (9 cm^2^) and kept overnight in a dust-free hood for drying. The composite film of biopolymers, thus obtained was stored at 4°C under dry conditions and was rinsed with phosphate buffer (0.1M, pH 8.0) before use (Tembe et al., 2006).

### Characterization of film through X-ray diffraction analysis

The powder X-ray patterns of the chitosan, gelatin and composite films were obtained using a Bruker D8 ADVANCE X-ray diffractometer, with Ni-filter and Cu-Kα radiation (λ = 1.544 Å) at a voltage of 40 kV and at 30 mA. Samples were scanned for relative intensity over the range of diffraction angle 2θ = 5–70°, with a scan speed of 1°/min at room temperature (Xu et al., 2006). Prior to testing, the samples were dried and stored in a hot air oven.

### Immobilization of enzyme

After washing with 0.1 M phosphate-buffered saline (pH 7.5), the carrier film was suspended in a tyrosinase solution at 4°C for overnight incubation. The unbound enzyme was removed by washing with 0.1 M sodium phosphate buffer (pH 8.0). Further, these films were rinsed with a 0.1 M phosphate-buffered saline (pH 7.5) to remove any unbound protein. The immobilized preparation was stored in the above phosphate buffer at 4°C till further use (Labus et al., 2011).

### Characterization of immobilization

#### Field emission scanning electron microscopy

The changes in surface structure and morphology of films consisting of chitosan, gelatin, chitosan-gelatin composite and composite film after immobilization of tyrosinase were observed using a Carl Zeiss Ultra Plus FESEM system (Germany) at an accelerating voltage of 10–20 kV.

#### Atomic force microscopy

To investigate the homogeneity of the films, the surface morphology of composite films before and after the immobilization of tyrosinase was observed using atomic force microscopy using NT-MDT (Moscow, Russia) multiple function unit. Different areas of control film and the film with immobilized enzyme were visualized under the same conditions. The average root mean square roughness of the film surface was collected using the topographical data obtained from AFM micrographs (Saini et al., 2014).

#### UV spectral analysis

Spectral changes with the free and immobilized enzyme were recorded in a range from 250 to 750 nm. For the assessment of monophenolase reaction of immobilized tyrosinase, films (9 cm^2^) were immersed in 3.0 ml of 0.5 M phosphate buffer solution (pH 6.5) containing 0.001 M L-tyrosine and 2% L-ascorbic acid for 10 min. All absorption spectra were recorded with CARY 100 Bio UV–Vis spectrophotometer (Agilent technologies, USA) at room temperature.

### Estimation of tyrosinase activity

The tyrosinase activity was determined by using L-tyrosine as substrate (Chen et al., 2001; Liu et al., 2004; Arica et al., 2007; Raval et al., 2012). For this the membrane with the immobilized enzyme (9 cm^2^) was kept in phosphate buffer (0.5 M, pH 6.5) containing 1 mM L-tyrosine with 2% L-ascorbic acid. Tyrosinase activity was estimated at 280 nm for 10 min using CARY 100 Bio UV–Vis spectrophotometer (Agilent technologies, USA). One unit of tyrosinase activity was equal to a ΔA_280_ of 0.001/min at 30°C in the reaction mixture containing L-tyrosine and L-ascorbic acid. All experiments were carried out in triplicate. The Y error bars in the figures indicate the standard error of the mean.

### Properties of free and immobilized enzyme

#### Thermostability and pH stability

A series of experiments were performed in order to characterize the immobilized enzyme response. Enzyme solution was freshly prepared every time. Thermal stability of both free and immobilized tyrosinase was determined by incubating the preparations at different temperatures i.e., 25–55°C for 60 min at constant pH. After incubation the preparation was left at room temperature for 60 min to equilibrate and was then assayed for enzyme activity as described above. All experiments were performed in triplicates and the relative enzyme activity was calculated by assigning the maximum activity as 100%.

The effect of pH on the activity of free and immobilized tyrosinase was studied in the pH range 4.0–8.0. For this two different buffer systems acetate buffer (for pH 4.0–5.0) and phosphate buffer (for pH 6.0–8.0) were employed. The optimum pH was determined by incubating the preparation at a given pH for 60 min at constant temperature. Then the pH of the preparation was adjusted to 7.0 and it was left for 60 min to equilibrate and then assayed for enzyme activity (Labus et al., 2011; Bayramoglu et al., 2013).

#### Loading efficiency, storage stability, and operational stability

For leaching studies, the ratio of the activities of tyrosinase adsorbed onto the membrane and the activity of enzyme before the immobilization was evaluated. To analyze the bound enzyme, membrane with immobilized enzyme was kept in assay buffer containing 1 mM L-tyrosine and 2% L-ascorbic acid for 10 min and the buffer was then decanted to a cuvette and the activity in the decanted solution was determined. The amount of enzyme that had leached out was calculated with the difference of initial enzyme activity and activity of adsorbed enzyme. Together they signify the percent of enzyme adsorbed onto the matrix and the enzyme that had been leached out (Munjal and Sawhney, 2002; Tembe et al., 2006).

$$\text{Loading efficiency }(\%)=\frac{\text{Activity of immobilized enzyme}}{\begin{array}{l} \text{Activity of free enzyme before}\\ \text{ immobilization} \end{array}}\times \text{​}100$$

Enzymes can easily be denatured and lose their catalytic activity. Therefore, operational and storage stabilities are essential parameters for an immobilized enzyme (Yildiz et al., 2013). The storage stabilities of the free and immobilized enzymes were investigated by storing in 0.1 M phosphate buffer (pH 7.0) at 4°C for a period of 20 days. Change in activity was measured at every 5th day for both the preparations.

The operational reusability of immobilized tyrosinase was examined by measuring the enzymatic activity of the same preparation successively for nine times using L-tyrosine as substrate. After each measurement, the membrane with immobilized enzyme was washed with phosphate buffer (0.1 M, pH 7.0) to remove any residual substrate and incubated in the same buffer for 1 h for equilibration. After that, the preparation was transferred into fresh reaction medium and enzyme activity was determined (Bayramoglu et al., 2013).

### L-DOPA production by immobilized enzyme

Partially purified and immobilized enzymes were evaluated for L-DOPA production by keeping the membrane in phosphate buffer (0.5 M, pH 6.5) containing 3 mg ml^−1^ L-tyrosine and 2% ascorbic acid. The amount of L-DOPA was determined according to the Arnow's method (Arnow, 1937). Briefly, 1 ml of above assay buffer was added with 1 ml of 0.5 M HCl and 1 ml of nitrate/molybdate reagent (giving yellow color) and then with 1 ml of 1 M NaOH (giving red color). Water was added to make a final volume to 5 ml. L-DOPA was estimated at 590 nm.

## Results

### Film characterization through X-ray diffraction analysis

**Figure 2:**
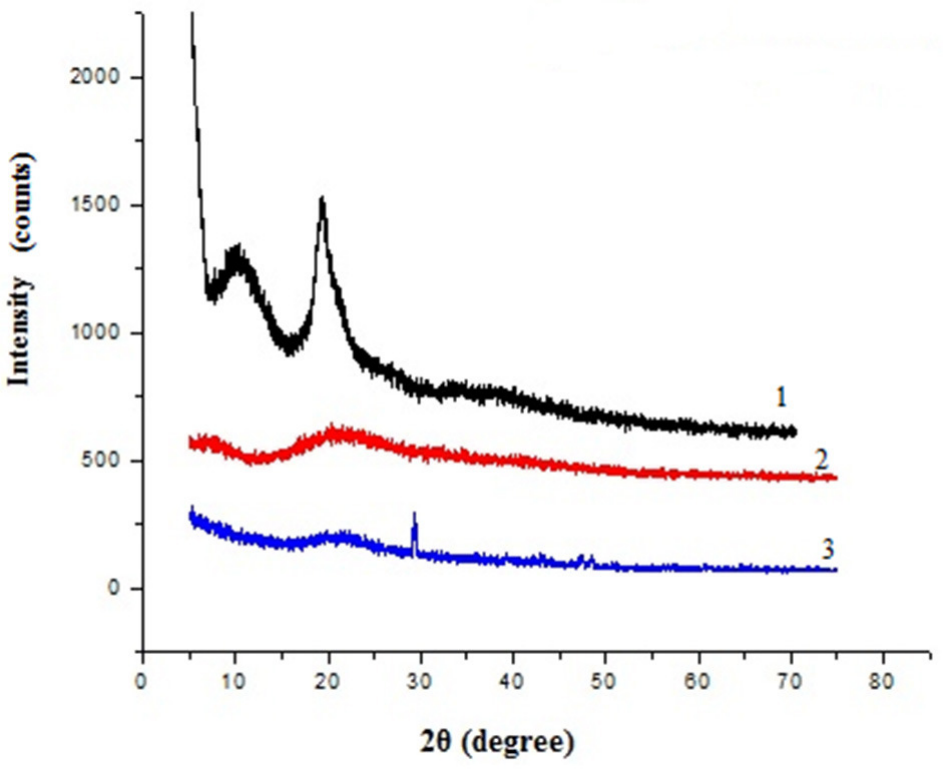
**Powder X-ray diffraction patterns of 1 chitosan, 2 gelatin, and 3 composite film**.

X-ray diffraction pattern of chitosan, gelatin and composite films were obtained and compared, which revealed marked differences in their structures (Figure 2). The diffraction pattern of chitosan exhibited distinct crystalline peaks at around 2θ values 10 and 20°, while XRD pattern of gelatin powder showed greater amorphous morphology with a characteristic broad hump in the range of 15–30° 2θ. It was also noticed that the crystalline peaks of chitosan disappeared as the composite was formed between chitosan and gelatin, instead a new very small peak was observed in the blend at 2θ around 29°. Also the broadened hump which was present in gelatin became lower in the composite film (15–25°), demonstrating an interaction between the two components.

### Characterization of immobilization

#### Field emission scanning electron microscopy

**Figure 3:**
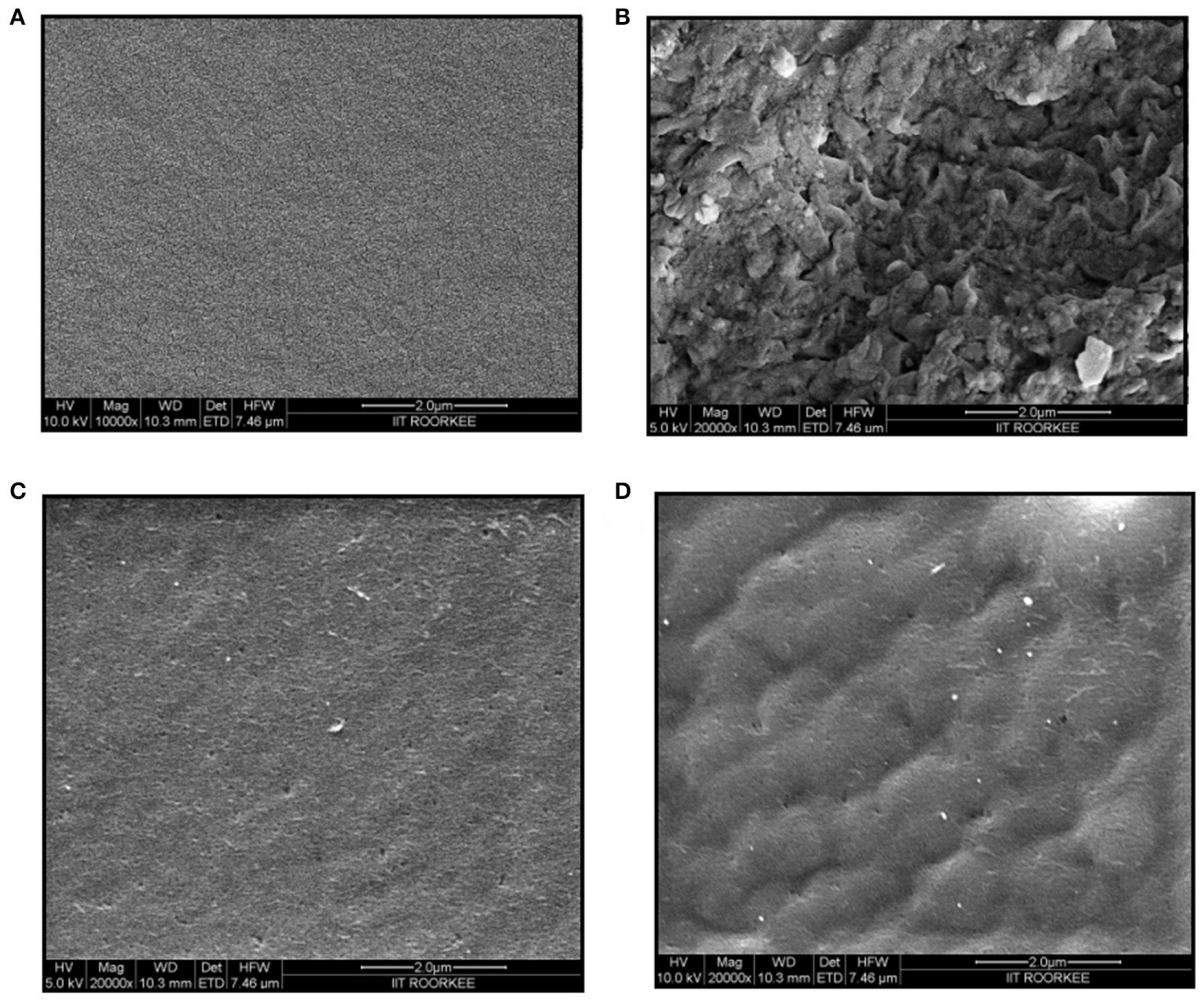
**FESEM images of chitosan film (A)**, gelatin film **(B)**, chitosan-gelatin composite film **(C)**, and composite film with immobilized tyrosinase **(D)**.

The physical morphologies of the chitosan, gelatin, chitosan-gelatin composite and tyrosinase immobilized hybrid films were observed by FESEM. The cross-sectional FESEM image of chitosan film exhibited many tiny pores, whereas gelatin film displayed regularly ordered particulate morphology (Figure 3). Chitosan-gelatin composite membrane is a cross-linked three-dimensional (3D) polymer network with a few smaller sphere particles filled in the mess space. When tyrosinase was adsorbed onto the film matrix, the network like structure disappeared and instead a regularly distributed aggregated smear was observed.

#### Atomic force microscopy

**Figure 4:**
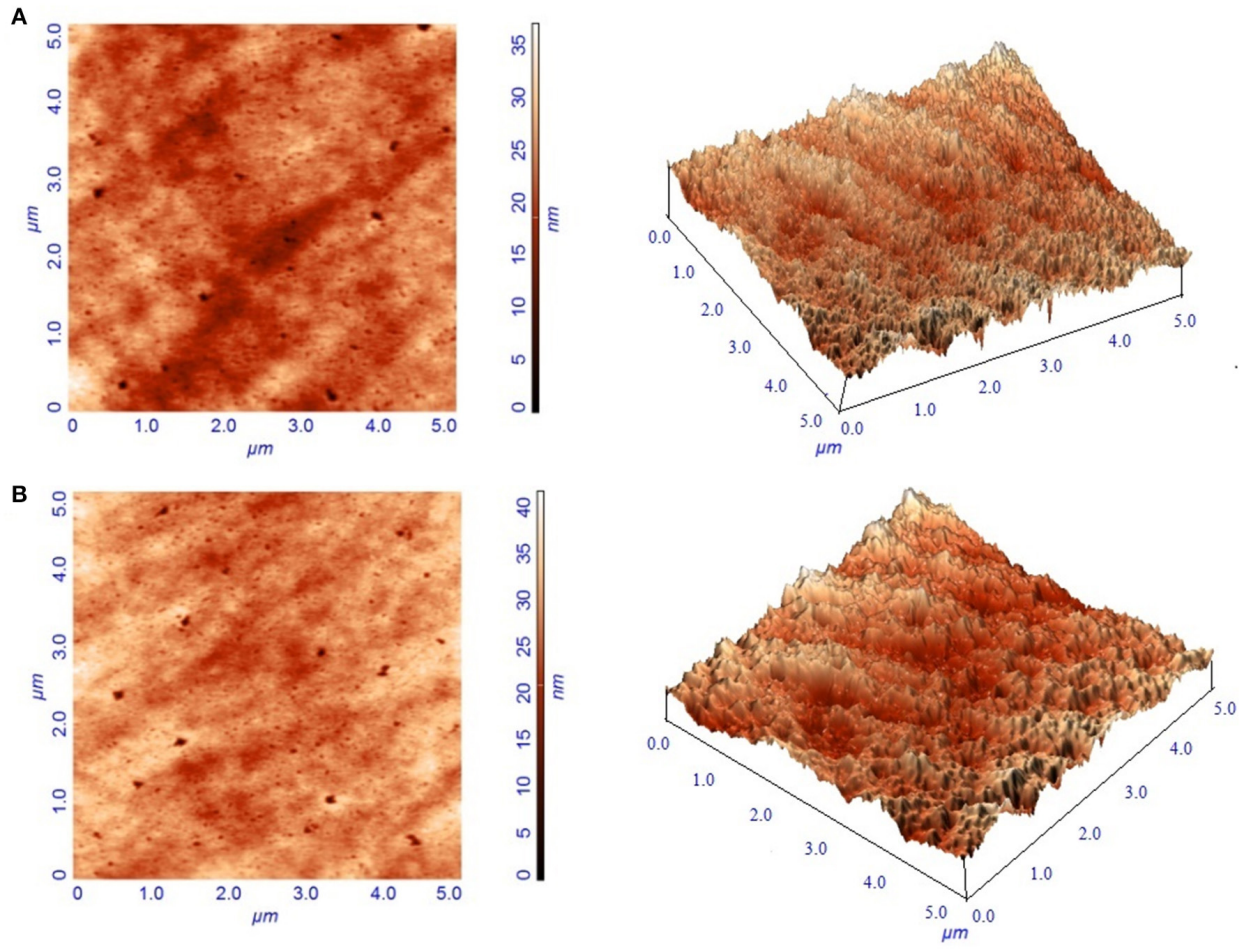
**Atomic force micrographs of blank composite film (A)** and film with immobilized tyrosinase **(B)**.

Immobilization of tyrosinase onto the composite film surface was also characterized by AFM micrographs. A typical AFM image of the surface of the chitosan-gelatin biocomposite membrane displayed a three-dimensional rough surface (Figure 4A). Tyrosinase immobilized matrix surface showed noticeable differences as compared to the control matrix (Figure 4B). The rough surface that was present on the blank film was more prominent after the adsorption of the enzyme onto its surface. For the blank film, the average root mean square roughness was observed to be 1.95 nm and after adsorption of the tyrosinase onto its surface, the value increased to 3.48 nm.

#### UV spectral analysis

**Figure 5:**
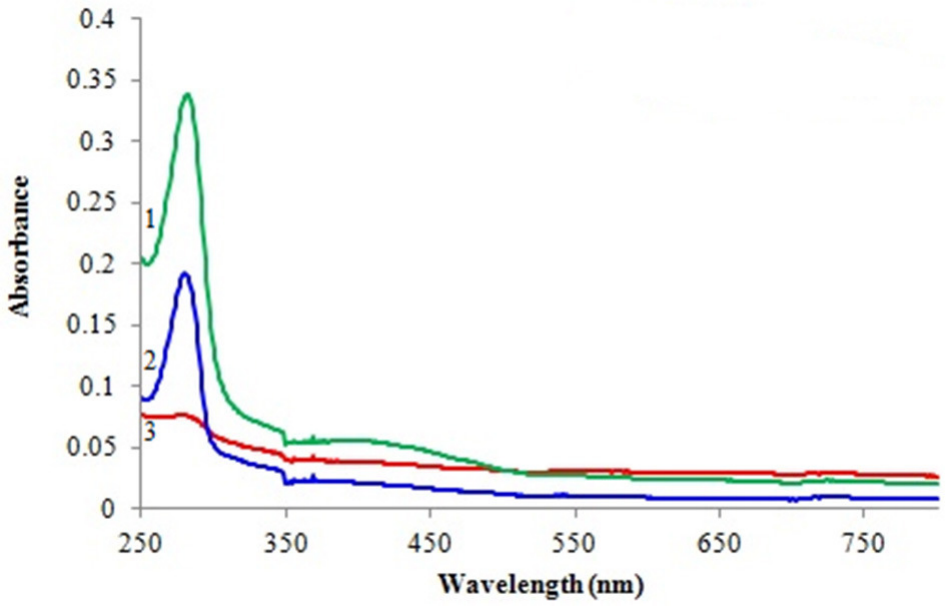
**Spectrophotometric analysis of L-DOPA with 1 free tyrosinase, 2 immobilized tyrosinase, and 3 blank film**.

Spectral changes during the course of reaction were recorded by performing wavelength scan (250–750 nm) for detection of L-DOPA. Figure 5 shows absorption spectra of the blank film, immobilized tyrosinase and free tyrosinase in the presence of L-tyrosine after 10 min of incubation. As evident from the spectra no change in absorbance was observed for the blank film, while free tyrosinase and film with immobilized tyrosinase had depicted absorption maxima at 280 nm.

### Properties of free and immobilized enzyme

#### Thermostability and pH stability

**Figure 6:**
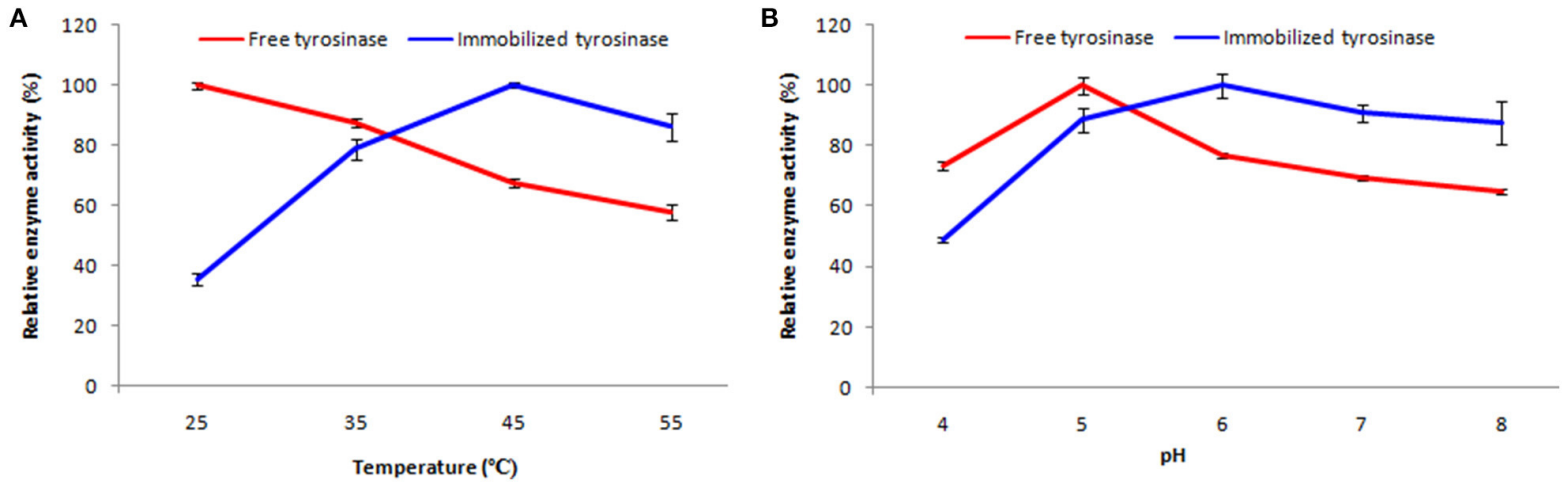
**Effect of temperature (A)** and pH **(B)** on percent activity of free and immobilized tyrosinase. The experiments were performed in triplicate and standard deviations are shown as error bars.

Soluble enzyme was most stable at 25°C and the stability decreased thereafter after increasing the temperature (Figure 6A). However, enzyme had 58% of the activity remaining following incubation at 55°C. Whereas, enzyme following immobilization appears to have increased thermostability, as only 14% loss of activity was observed at 55°C following immobilization. Relative enzyme activity was calculated by assigning the maximum activity value as 100%.

Free enzyme was observed to be most active at pH 5.0 with a quick drop in its activity at acidic and basic pH, while the pH profile of the immobilized tyrosinase displayed comparatively better activity in a broader pH range (5.0–8.0) with maximum activity at pH 6.0 (Figure 6B).

#### Loading efficiency, storage stability, and operational stability

As observed about 72% retention of activity was observed following immobilization as compared to the free enzyme preparation, thus portraying the immobilization to be reasonably effective.

**Figure 7:**
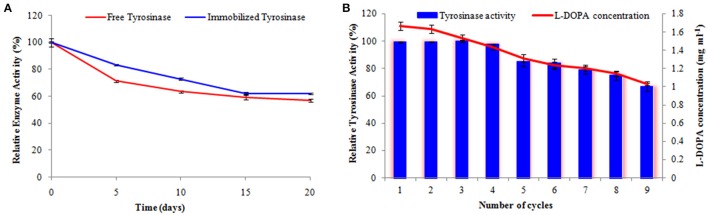
**Activity of free and immobilized tyrosinase during the course of storage (A)** and retention in enzyme activity and L-DOPA production following repetitive cycles **(B)**. The experiments were performed in triplicate and standard deviations are shown as error bars.

The activity of the immobilized tyrosinase decreased more slowly compared to the free tyrosinase under the similar storage conditions. Free tyrosinase retained only 71% of its original activity after 5 days of incubation while immobilized enzyme was observed to have 84% activity. Free enzyme lost almost 40% of its initial activity within 10 days, whereas the immobilized enzyme maintained more than 72% of its original activity after 10 days, when stored at 4°C (Figure 7A).

Operational stability of enzyme-composite preparation was evaluated following 9 repetitive cycles (Figure 7B). The immobilized tyrosinase maintained 97% of its activity and produced 1.44 mg ml^−1^ L-DOPA until 4th cycle and retained 84% of its activity and produced 1.23 mg ml^−1^ L-DOPA even following six successive cycles.

### L-DOPA production

Addition of nitrite-molybdate reagent to the buffer with immobilized enzyme led into the formation of yellow color. Further addition of sodium hydroxide resulted into the development of a red color, similar to the one developed with partially purified enzyme. L-DOPA levels obtained were 1.9 mg ml^−1^ and 1.7 mg ml^−1^ with partially purified and immobilized enzyme, respectively (Table 1). The L-DOPA production declined to 55% after 48 h and then to 27% following 72 h of storage with partially purified enzyme, while immobilized enzyme still had resulted into 88% and 56% L-DOPA production after 48 h and 72 h of storage, respectively.

**Table 1:** **L-DOPA production with free and immobilized tyrosinase**.

**Time (h)**	**L-DOPA production (mg ml^−1^)**	
	Free Enzyme^*^	Immobilized Enzyme^*^
24	1.901 ± 0.012	1.701 ± 0.050
48	1.054 ± 0.021	1.491 ± 0.021
72	0.504 ± 0.050	0.954 ± 0.026

* *The experiments were performed in triplicate and standard deviations are shown as error bars*.

## Discussion

Tyrosinase from *A. niger* PA2 has been immobilized onto a bio-composite film consisting of chitosan and gelatin polymers. The homogeneity of the film was characterized by X-ray diffraction analyses (Figure 2). The distinct crystalline peaks observed in the diffraction pattern of chitosan were due to the abundance of −OH and −NH_2_ groups in the chitosan structure, which could form strong inter and intra molecular hydrogen bonds and thus the chitosan molecules form crystalline regions and gain certain regularity easily (Ramya et al., 2012). While a broad hump observed in XRD pattern of gelatin powder was due to its amorphous morphology. It was also observed that the crystalline peaks of chitosan disappeared as the composite was formed between chitosan and gelatin, which is due to the elimination of hydrogen bonding between amino groups and hydroxyl groups in chitosan (Smitha et al., 2005). Also the broadened hump which was present in gelatin became lower in the composite film, demonstrating an interaction between the two components. To evaluate immobilization efficiency, chitosan-gelatin-Tyr bio-composite films were analyzed by FESEM, AFM and UV-spectroscopy. As observed through FESEM, chitosan-gelatin composite membrane is a cross-linked three-dimensional network with a few smaller sphere particles filled in the mess space (Figure 3). This network like structure could contribute a significant role toward the high enzyme stability within the polymer matrix providing a large surface area for efficient immobilization of enzyme (Liu et al., 2005). When tyrosinase was immobilized onto the film matrix, the network like structure disappeared and instead a regularly distributed aggregated smear was observed. A typical AFM image of the surface of the chitosan-gelatin bio-composite membrane displayed a three-dimensional rough surface (Figure 4), which provided a significantly larger surface area, improving its loading efficiency for the immobilization of biomolecules and other substances and also effectively preventing the leaking of biomolecule from the preparation. The roughness of the film surface is an important aspect that may provide information about the surface modifications after immobilization. It was apparent that the surface of the blank film is less rough than that of immobilized preparation. Increase in the roughness value after adsorption of the enzyme suggests the deposition of enzyme molecules onto the matrix surface (Saini et al., 2014). UV-spectral analysis also depicted the formation of L-DOPA, when the film with immobilized enzyme was immersed in buffer containing L-tyrosine (Figure 5). Soluble enzyme was most stable at 25°C and the stability decreased thereafter after increasing the temperature (Figure 6A), whereas, enzyme following immobilization appears to have increased thermostability at higher temperature i.e., upto 55°C. Munjal and Sawhney (2002) obtained similar results with gelatin gel. They observed temperature optima for free tyrosinase from mushroom at 20°C, while the tyrosinase immobilized onto gelatin gel demonstrated the optima at 40°C. Labus et al. (2011) observed temperature maxima for *Agaricus bisporus* tyrosinase around 15–25°C, while enzyme immobilized onto cellulose based carrier had shown maxima around 30–40°C. Bayramoglu et al. (2013) obtained the optimum reaction temperature for the immobilized tyrosinase onto biosilica at 45°C, which was 10°C higher than its free counterpart. The comparatively higher thermostability of the immobilized enzyme as compared to the soluble enzyme may possibly be due to its conformational rigidity due to the enzymatic interactions with the matrices (Labus et al., 2011; Bayramoglu et al., 2013) and thus preventing the unfolding (Mateo et al., 2007; Zhang et al., 2011). pH is one of the vital factors influencing the amino acid nature in the protein structure. Free enzyme was observed to be most active at pH 5.0 with a quick drop in its activity at acidic and basic pH. While the pH profile of the immobilized tyrosinase displayed comparatively better activity in a broader pH range (Figure 6B). Labus et al. (2011) observed pH optima with tyrosinase immobilized onto cellulose based carrier around pH 7.0–9.0, while free tyrosinase was most active around pH 6.0–7.0. These results implied that the immobilization led to retention of the enzyme activity at extreme pH due to adsorption of tyrosinase within the support matrix (Monsan, 1978; Bayramoglu and Arica, 2010; Li et al., 2012), thus making this biocatalyst more suitable for industrial exploitation (Bayramoglu et al., 2013). For leaching studies, the ratio of the activities of tyrosinase adsorbed onto the membrane and the activity of free enzyme i.e., before immobilization was calculated. About 72% of activity was retained following immobilization as compared to the free counterpart. It was also expected beforehand that immobilized/free tyrosinase activity ratio should be lower than 1.0 (Labus et al., 2011). Thus, this matrix proves to be a biocompatible composite that provides a natural environment to the enzyme, minimizing it's leaching, retaining the activity and therefore making the immobilization highly productive. Generally there is a gradual decrease in enzyme activity upon storage and immobilization significantly slows down this phenomenon (Figure 7A). The activity of the immobilized tyrosinase decreased more slowly compared to the free tyrosinase under the similar storage conditions. Immobilized enzyme was observed to have 84% activity after 5 days of incubation and was able to maintain more than 72% of its original activity after 10 days, which indicates that the immobilization of the enzymes decreases the likelihood of its denaturation (Zhang et al., 2011). Munjal and Sawhney (2002) also obtained 65–75% retention in enzyme activity with tyrosinase immobilized onto polyacrylamide gel after 14 days of incubation. Bayramoglu et al. (2013) had also observed 80% retention of activity after 14 days of incubation with tyrosinase immobilized onto biosilica. The greater stability of the immobilized enzyme may also be ascribed as an effect of film (Munjal and Sawhney, 2002), which provides a biocompatible microenvironment to the enzyme (Schnapp and Shalitin, 1976). These results are also in agreement with the previous studies done by several groups on enzyme immobilization (Edwards et al., 1999; Bayramoglu et al., 2011; Karagoz et al., 2011; Nicolucci et al., 2011). The reproducibility of the immobilized enzyme preparation is an important attribute for large-scale applications. The immobilized tyrosinase maintained 84% of its activity and produced 1.23 mg ml^−1^ L-DOPA even following six successive cycles (Figure 7B). Munjal and Sawhney (2002) also obtained 75–85, 60–70, and 45–50% retention in enzyme activity after 5 cycles of reuses with tyrosinase immobilized onto alginate, polyacrylamide and gelatin gel, respectively, whereas, Labus et al. (2011) observed 45–65% retention in *Agaricus bisporus* tyrosinase activity after 6 reuses when it was immobilized onto cellulose based carriers. Further 80% of the enzyme activity was retained following 7 cycles with tyrosinase immobilized onto polystyrene microplate (Saini et al., 2014). Higher retention of enzyme activity and ability to produce L-DOPA following repetitive usage may be attributed to the effective adsorption of enzyme onto the polymer matrix, thus making it promising and amenable for consistent usage for industrial exploitations.

## Conclusions

In the present study, tyrosinase from a fungal source, *A. niger* PA2 was immobilized onto a composite of two biopolymers: chitosan and gelatin. This is the first report where tyrosinase extracted from *A. niger* was immobilized onto chitosan-gelatin copolymer matrix and evaluated for its activity and L-DOPA synthesis. Being biocompatible, this unique composite film provided a suitable natural microenvironment, which could successfully offer efficient loading and thus prevent the leaching of the immobilized enzyme. The enzyme adsorbed onto the co-polymer matrix exhibited notably increased thermal and pH stability. The immobilized preparation was stable for 20 days and could be reused for multiple cycles. The reusability of enzyme-co-polymer system may provide cost-effective strategy for large-scale production of L-DOPA. Thus, the proposed blend of the enzyme-biocomposite film may provide a promising and viable system for L-DOPA production and for a wide range of biotechnological applications.

## Author contributions

All authors listed, have made substantial, direct and intellectual contribution to the work, and approved it for publication.

### Conflict of interest statement

The authors declare that the research was conducted in the absence of any commercial or financial relationships that could be construed as a potential conflict of interest.
